# Supplementary material for: Nucleosome fibre topology guides transcription factor binding to enhancers
Source: Nature. 2024 Dec 18;638(8049):251–60. doi: 10.1038/s41586-024-08333-9 (PMC11798873; doi:10.1038/s41586-024-08333-9)
Supplement: Supplementary file 1 — Supplementary Figs. 1 and 2 and Supplementary Tables 2–4. [file 41586_2024_8333_MOESM1_ESM.pdf]

nature portfolio

https://doi.org/10.1038/s41586-024-08333-9

Supplementary information

Nucleosome fibre topology guides transcription factor binding to enhancers

In the format provided by the authors and unedited

Supplementary Figure 1:

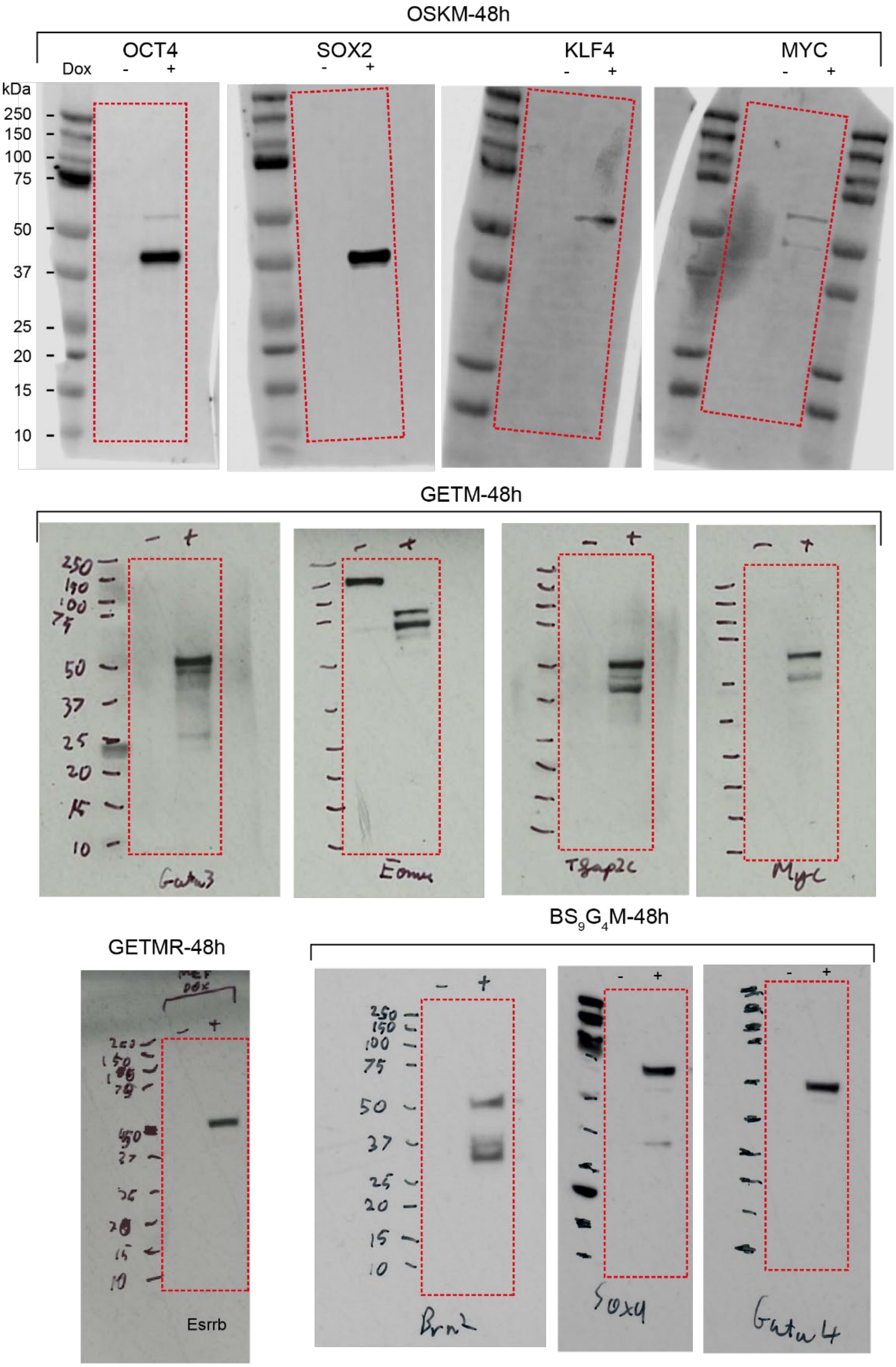

Unprocessed data for Western blots shown in Extended Data Fig. 1d. Red boxes indicate where the images were cropped.

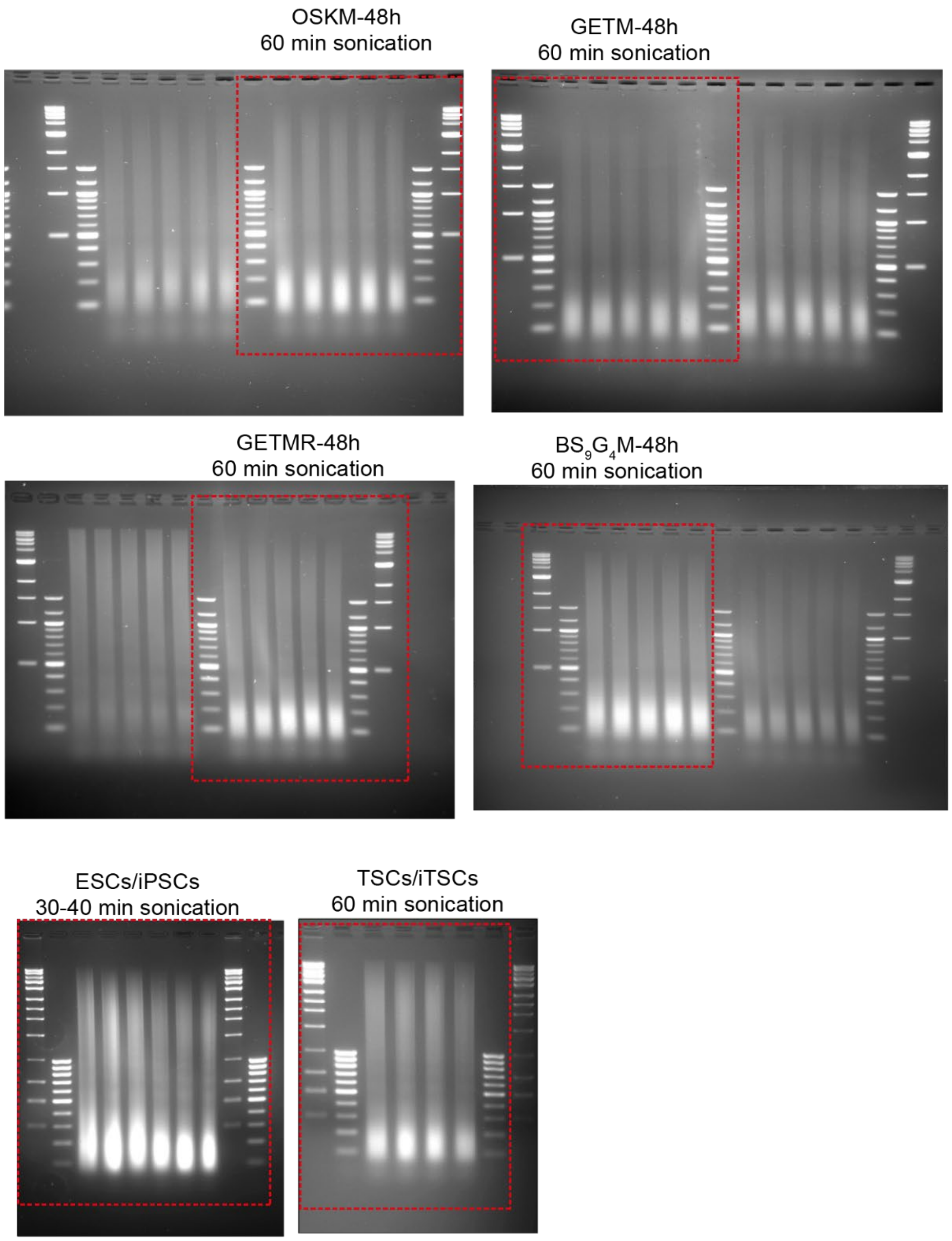

Unprocessed data for agarose gels shown in Extended Data Fig. 1e. Red boxes indicate the cropping of the images.

MEFs

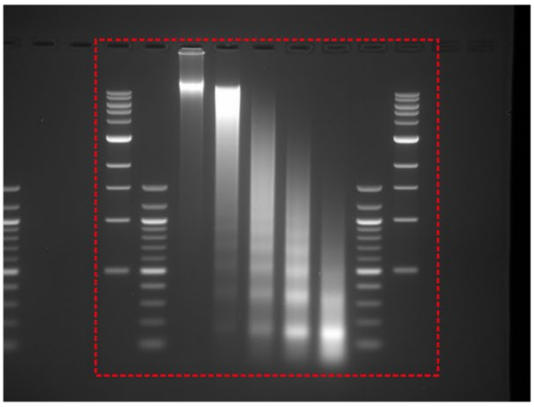

iPSCs

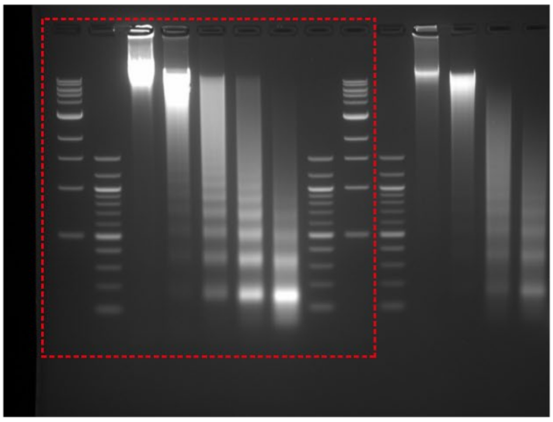

iTSCs

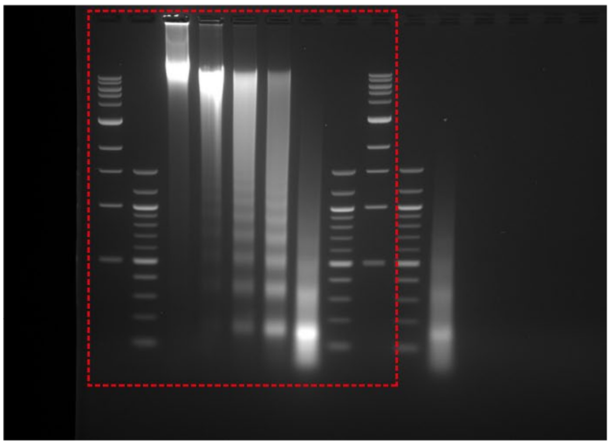

Unprocessed data for agarose gels shown in Extended Data Fig. 3a. Red boxes indicate the cropping of the images.

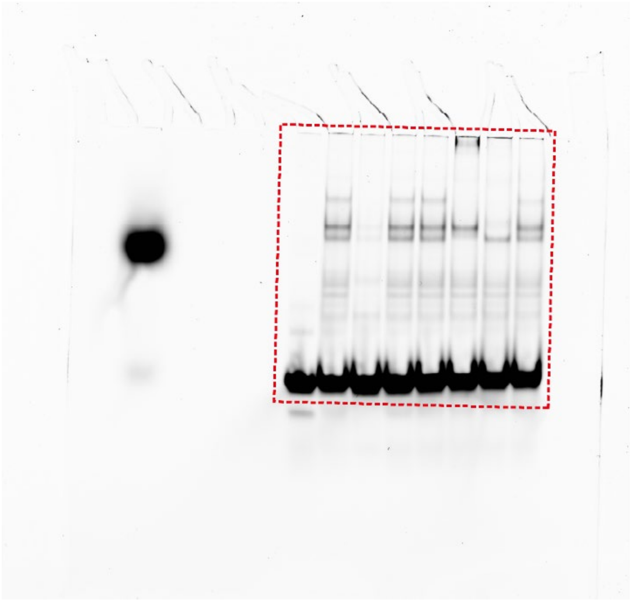

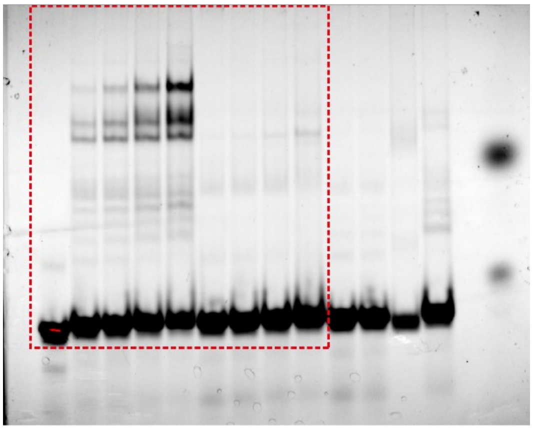

Unprocessed data for EMSAs shown in Extended Data Fig. 5c,d. Red boxes indicate the cropping of the images.

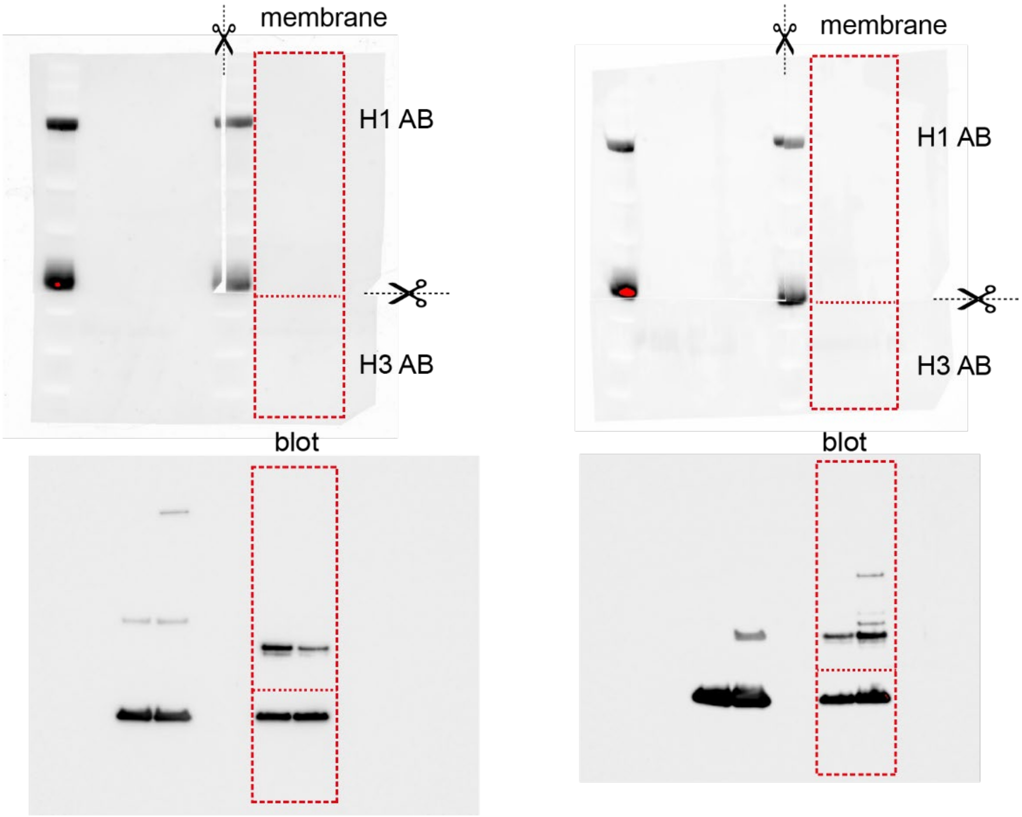

Unprocessed data for Western blots shown in Extended Data Fig. 7j. Red boxes indicate the cropping of the images.

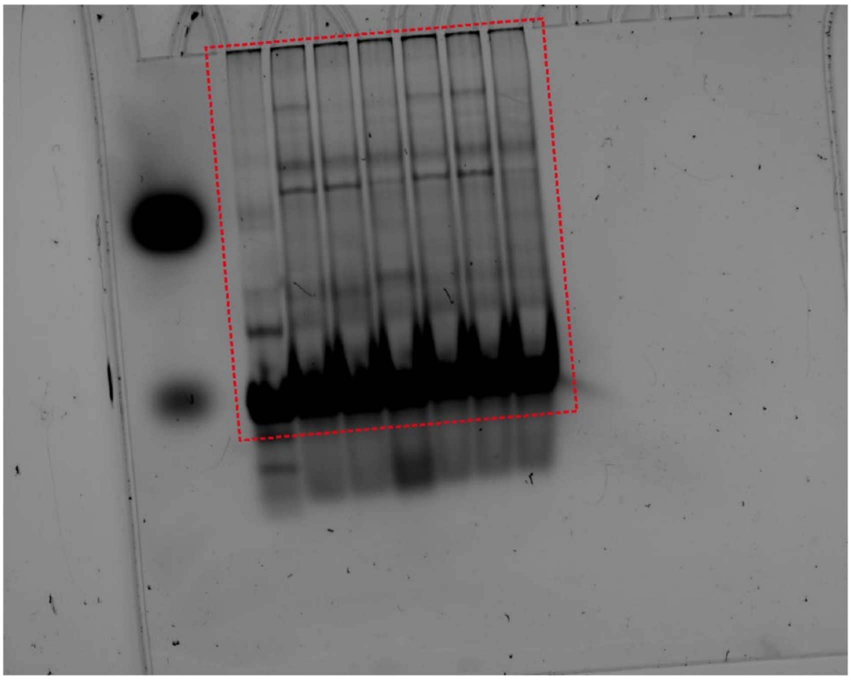

Unprocessed data for EMSAs shown in Extended Data Fig. 9g. Red boxes indicate the cropping of the images.

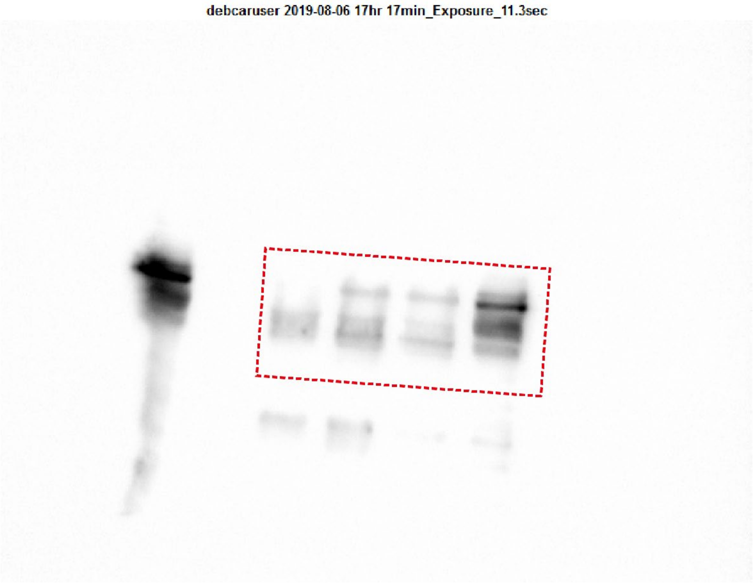

Unprocessed data for Western blots shown in Extended Data Fig. 9h. Red boxes indicate the cropping of the images.

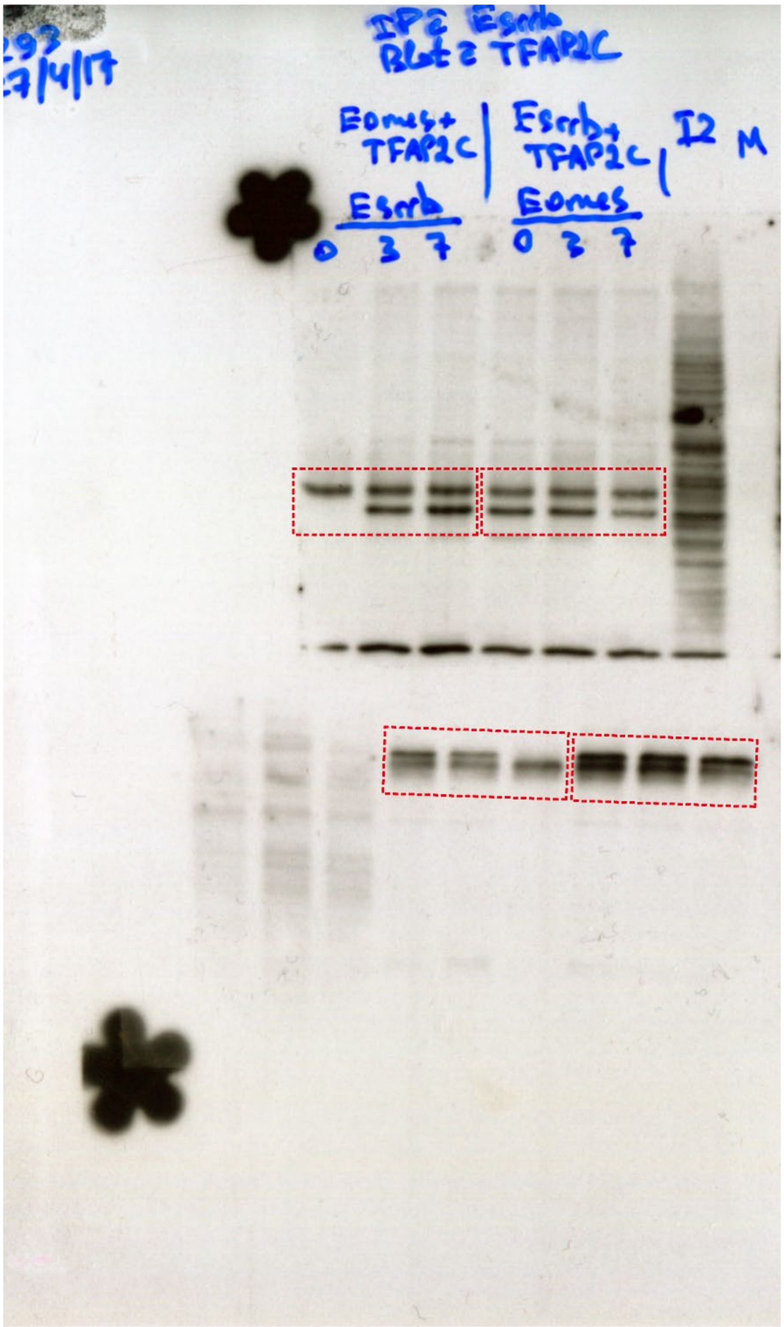

Unprocessed data for Western blots shown in Extended Data Fig. 10g. Red boxes indicate the cropping of the images.

Supplementary Figure 2:

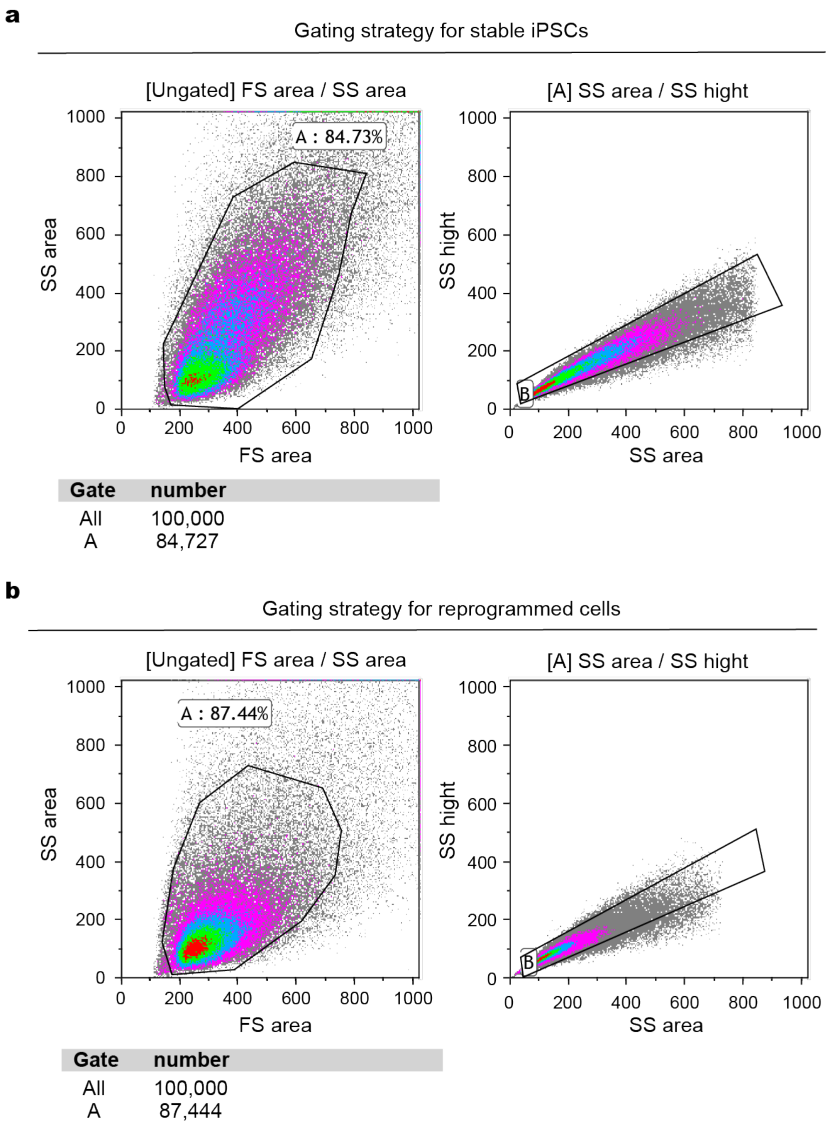

Example of gating strategy of stable iPSCs (a) and reprogrammed cells (b).

Supplementary Table 2: Antibodies used in this study.

Antibody	Supplier	Product Code	Immunostaining dilution (µg/ml)	Western Blotting dilution (µg/ml)	Used for ChIP
OCT4	Abcam	ab19857	2.5 (48hr)	1	Yes
OCT4	Abcam	ab27985	5(iPSCs)	n/a	n/a
SOX2	R&D	AF2018	5 (48hr)	0.45	Yes
SOX2	Abcam	ab97959	5 (iPSCs)	n/a	n/a
Mouse KLF4	R&D	AF3158	n/a	n/a	Yes
Human KLF4	R&D	AF3640	5	0.2	Yes
MYC	R&D	AF3696	10	0.66	Yes
GATA3	R&D	AF2605	10	0.66	Yes
EOMES	Abcam	ab23345	1.6	1.4	Yes
TFAP2C	R&D	AF5059	0.75	1	Yes
BRN2	Santa-Cruz	sc-6029-X	2	0.5	Yes
SOX9	Millipore	AB5535	1	1	Yes
GATA4	Santa-Cruz	sc-1237-X	2	0.5	Yes
ESRRB	Proteintech	22644-1-AP	5	n/a	No
ESRRB	R&D	PP-H6705-00	n/a	1	Yes
NANOG	Bethyl	A300-397A	5	n/a	n/a
CDX2	Biogenex	CDX2-88	1:500	n/a	n/a
H1.4	Merck	H7665	n/a	1.6	n/a
Pan-H1	Active Motif	AB_2793314	n/a	1:1000 dilution of stock	n/a
H3	Abcam	ab24824	n/a	0.2	n/a
Donkey Anti-Goat IgG-HRP	Santa-Cruz	sc-2020	n/a	0.8	n/a
Goat Anti-Mouse IgG-HRP	Santa-Cruz	sc-2005	n/a	0.8	n/a
Goat Anti-Rabbit IgG-HRP	Santa Cruz	sc-1004	n/a	0.8	n/a
Goat Anti-Rabbit Alexa Fluor 488	Abcam	ab150077	4	n/a	n/a
Donkey Anti-Goat Alexa Fluor 488	Abcam	ab150129	4	n/a	n/a

Supplementary Table 3: DNA constructs used in this study.

**Insert**	**Organism**	**Plasmid backbone**	**Addgene #**	**Reference**
eGFP	*Aequorea victoria*	pWPT	n/a	Soufi et al (2012)
rTTA2-M2	n/a	pWPT	n/a	Kim et al (2013)
OCT4	*Homo sapiens*	pFUW-TetO	20726	Hockemeyer et al (2008)
SOX2	*Homo sapiens*	pFUW-TetO	20724	Hockemeyer et al (2008)
KLF4	*Homo sapiens*	pFUW-TetO	20725	Hockemeyer et al (2008)
HA-c-MYC	*Homo sapiens*	pFUW-TetO	20723	Hockemeyer et al (2008)
GATA3	*Homo sapiens*	pFUW-TetO	n/a	This study
EOMES	*Homo sapiens*	pFUW-TetO	n/a	This study
TFAP2C	*Homo sapiens*	pFUW-TetO	n/a	This study
ESRRB	*Homo sapiens*	pFUW-TetO	n/a	This Study
Brn2	*Mus musculus*	pFUW-TetO	27151	Vierbuchen et al (2010)
Sox9	*Mus musculus*	pFUW-TetO	41080	Buganim et al (2012)
Gata4	*Mus musculus*	pFUW-TetO	41084	Buganim et al (2012)
H1.4	Homo sapiens	pFUW-TetO	n/a	This study
shRNA to Hist1h1e	*Mus musculus*	pLKO.1	n/a	Moffat et al (2006)
empty	*n/a*	pLKO.1	#8453	Stewart et al (2003)
n/a	*n/a*	psPAX2	12260	Trono Lab (unpublished)
n/a	*n/a*	pMD2.G	12259	Trono Lab (unpublished)
Nanog_enh-eGFP and Nanog_flip-tdTomato	*Mus musculus*	PB-TAP	n/a	This study
piggyBac transposase	*Trichoplusia ni*	pCMV	n/a	Yusa et al (2011)

Supplementary Table 4: Primer list used for qPCR

Gata3^{transgenic}	FWD: 5'-CATCCCTGAGCCACATCTCT-3'
Eomes ^{transgenic}	FWD: 5'-CCCCCATAAAGTGTGAGGAC-3'
Tfap2c ^{transgenic}	FWD: 5'-AGGAGGCTCTAATCGCCATC-3'
Myc ^{transgenic}	FWD: 5'-TGTCCATTCAAGCAGACGAG-3'
Esrrb ^{transgenic}	FWD: 5'-AGCCGTGCAACACTTCTACA-3'
Sox2 ^{transgenic}	FWD: 5’-GCCCAGTAGACTGCACATGG-3’
FUW-vector-WPRE ^{transgenic}	RVS: 5'-GGCATTAAAGCAGCGTATCC-3'
Oct4 ^{transgenic}	RVS: 5’-GTTGGTTCCACCTTCTCCAA-3’
Klf4 ^{transgenic}	RVS: 5’-ACGCAGTGTCTTCTCCCTTC-3’
FUW-vecor-tetO ^{transgenic}	FWD: 5’-CGCCTGGAGACGCCATCCACGCT-3’
