# Supplementary material for: Nucleosome fibre topology guides transcription factor binding to enhancers
Source: Nature. 2024 Dec 18;638(8049):251–60. doi: 10.1038/s41586-024-08333-9 (PMC11798873; doi:10.1038/s41586-024-08333-9)
Supplement: Supplementary file 2 — Reporting Summary [file 41586_2024_8333_MOESM2_ESM.pdf]

nature portfolio

Corresponding author(s): Abdenour Soufi

Last updated by author(s): Sep 30, 2024

Reporting Summary

Nature Portfolio wishes to improve the reproducibility of the work that we publish. This form provides structure for consistency and transparency in reporting. For further information on Nature Portfolio policies, see our Editorial Policies and the Editorial Policy Checklist.

Statistics

For all statistical analyses, confirm that the following items are present in the figure legend, table legend, main text, or Methods section.

n/a	Confirmed	
☐	☒	The exact sample size (*n*) for each experimental group/condition, given as a discrete number and unit of measurement
☐	☒	A statement on whether measurements were taken from distinct samples or whether the same sample was measured repeatedly
☐	☒	The statistical test(s) used AND whether they are one- or two-sided<br>*Only common tests should be described solely by name; describe more complex techniques in the Methods section.*
☐	☒	A description of all covariates tested
☐	☒	A description of any assumptions or corrections, such as tests of normality and adjustment for multiple comparisons
☐	☒	A full description of the statistical parameters including central tendency (e.g. means) or other basic estimates (e.g. regression coefficient) AND variation (e.g. standard deviation) or associated estimates of uncertainty (e.g. confidence intervals)
☐	☒	For null hypothesis testing, the test statistic (e.g. *F*, *t*, *r*) with confidence intervals, effect sizes, degrees of freedom and *P* value noted<br>*Give P values as exact values whenever suitable.*
☐	☒	For Bayesian analysis, information on the choice of priors and Markov chain Monte Carlo settings
☐	☒	For hierarchical and complex designs, identification of the appropriate level for tests and full reporting of outcomes
☐	☒	Estimates of effect sizes (e.g. Cohen's *d*, Pearson's *r*), indicating how they were calculated

Our web collection on statistics for biologists contains articles on many of the points above.

Software and code

Policy information about availability of computer code

Data collection

- Sequencing data was collected using the Illumina 2500, 4000 and NovaSeq platforms.
- Western blots and EMSAs were imaged using BioRAD ChemiDoc MP imaging system.
- Fluorescent immunostaining images were captured by Nikon Eclipse T! microscope and IRIS Digital Cell Imaging System.
- DNA fragment sizes were measure by Agilent 2200 Tapestation.
- Histones were analyzed by reversed-phase high pressure liquid chromatography using Waters 2695 system equipped with a Vydac 218TP C18 HPLC column, and Waters 996 Photodiode Array Detector.
- LCMS analyses were performed on a TripleTOF 5600+ mass spectrometer (AB SCIEX) coupled with M5 MicroLC system (AB SCIEX/Eksigent) and PAL3 autosampler.
- For colony counting whole wells were imaged at a resolution of 4 µm/pixel using a CELIGO image cytometer.
- For flow cytometery, a Beckman Coulter (Gallios) flow cytometer was used. Data acquisition and analysis were conducted using the Kaluza Software (Version 1.0.14029.14028).

Data analysis

data was analyzed using the following open source and commercial softwares: FASTQC v0.11.8, Bowtie v2.3, Bedtools V2.28, Picard v2.20, MACS v2.1.1, DeepTools V2, Qhttps://git.ecdf.ed.ac.uk/soufi_lab/motif_mononucleosomeualimap V2.2.1, SAMTool2 v1.3.1, BEDOPS V2.49, MEME v5.0.2, R v3.6 (various R packages as indicated in the methods), Cutadapt v3.3, STAR 2.7, DANPOS2, plot2DO v1.0, cLoops2, Coolpup.py v0.9.7, Pymol v3.0.3 with APBS plugin, IGV version 2.13.2 run with JAVA 11.0.13 (OpenJDK 64-bit). AlphaFold Multimer (AlphaFold2 version 2.3.1 on COSMIC2 cloud server), ImageJ (v1.54f) and Java 1.8.0_322 (64-bit), PeakView (version 2.2.0.11391, ABSciex), MicroApp (version 2.0.1.2133, ABSciex), UpSetR (version 1.4.0), FitHiChIP (version 11.0).

Custom scripts were deposited in UOE GitHub:

<https://git.ecdf.ed.ac.uk/soufi_lab/motif_mononucleosome>
<https://git.ecdf.ed.ac.uk/soufi_lab/motif_nucleosome_arrays>

For manuscripts utilizing custom algorithms or software that are central to the research but not yet described in published literature, software must be made available to editors and reviewers. We strongly encourage code deposition in a community repository (e.g. GitHub). See the Nature Portfolio guidelines for submitting code & software for further information.

Data

Policy information about availability of data

All manuscripts must include a data availability statement. This statement should provide the following information, where applicable:

- Accession codes, unique identifiers, or web links for publicly available datasets
- A description of any restrictions on data availability
- For clinical datasets or third party data, please ensure that the statement adheres to our policy

All next generation sequencing data generated as part of this study have been deposited in the Gene Expression Omnibus (GEO) under the series accession number GSE201852 (released upon publication, reviewers' access token: izkngkyynzutvmv). Previously published H3K27ac ChIP-seq, RNA-seq and ATAC-seq data were obtained from GSE98124, GSE171127 and GSE70234. Histone H1 ChIP-seq was obtained from GSE156697 and GSE46134. Oct4 ChIP-seq in MEFs-Oct4-48h were obtained from GSE168142. CTCF, PolII, P300, H3K4me1/3 ChIP-seq from GSE29184 and GSE29218. H3K9me1/me2 ChIP-seq from GSE54412. Rad21 ChIP-seq from GSE111820 and GSE115984. Brn2 ChIP-seq from GSE35496. HP1α, Suv39h1/2, and H3K9me3 ChIP-seq are from GSE57092. Oct4 and Sox2 ChIP-seq from secondary OSKM reprogramming system obtained from GSE101905. OSKM ChIP-seq in Mbd3f/- secondary reprogramming system are obtained from GSE102518. All data aligned to mouse reference genome MGSCv37 (mm9).

Research involving human participants, their data, or biological material

Policy information about studies with human participants or human data. See also policy information about sex, gender (identity/presentation), and sexual orientation and race, ethnicity and racism.

Reporting on sex and gender	N/A
Reporting on race, ethnicity, or other socially relevant groupings	N/A
Population characteristics	N/A
Recruitment	N/A
Ethics oversight	N/A

Note that full information on the approval of the study protocol must also be provided in the manuscript.

Field-specific reporting

Please select the one below that is the best fit for your research. If you are not sure, read the appropriate sections before making your selection.

☒ Life sciences ☐ Behavioural & social sciences ☐ Ecological, evolutionary & environmental sciences

For a reference copy of the document with all sections, see nature.com/documents/nr-reporting-summary-flat.pdf

Life sciences study design

All studies must disclose on these points even when the disclosure is negative.

Sample size	Sample sizes are indicated in each Figure legend. Generally, three to six biological replicates were used. This was determined according to established methods in the field and previous experience such as that in (Soufi et al; DOI:10.1016/j.cell.2012.09.045 and Soufi et al; DOI:10.1016/j.cell.2015.03.017), which allowed us to predetermine the number of sample size of each experiment.<br>The number of ChIP-seq peaks were identified to be significantly enriched over input by MACS2 software (q = 0.01). ATAC-seq enrichment within was considered significant based on 20 normalized reads or more. All sequencing libraries were normalized to sequencing depth.
Data exclusions	All replicates and no data were excluded. All experiment included positive, negative or internal controls. To remove over-represented sequencing data, ENCODE black list were excluded from all next-generation sequencing data. Sequencing duplicated reads generated by PCR and optical density and non-uniques sequences were removed from all next-generation sequencing data to remove PCR bias.
Replication	All replication attempt were successful. Each Experiment was repeated as indicated in the figure legends and the Methods section.
Randomization	For pileup Micro-C, 10 random control regions located between 100 kb and 1Mb away per interval per averaged window were used. Random sequences with similar group number and size for nucleosome array were generated using Bedtools shuffle. Motif analysis used random sequences that are 1kb away from ChIP-seq peaks.

All reprogramming experiments included non-reprogrammed controls or infected controls with empty vectors so so each experimental condition can be allocated into separate groups.

Blinding

H1-KO and H1-OE for ATAC-seq and Micro-C were blinded.
Sequencing data showed the same diversity and were normalized to the sequencing depth. No blinding was required when bias can be quantified and removed.

Reporting for specific materials, systems and methods

We require information from authors about some types of materials, experimental systems and methods used in many studies. Here, indicate whether each material, system or method listed is relevant to your study. If you are not sure if a list item applies to your research, read the appropriate section before selecting a response.

Materials & experimental systems

n/a	Involved in the study	
☐	☒	Antibodies
☐	☒	Eukaryotic cell lines
☒	☐	Palaeontology and archaeology
☐	☒	Animals and other organisms
☒	☐	Clinical data
☒	☐	Dual use research of concern
☒	☐	Plants

Methods

n/a	Involved in the study	
☐	☒	ChIP-seq
☐	☒	Flow cytometry
☒	☐	MRI-based neuroimaging

Antibodies

Antibodies used

All antibodies and amounts are listed in Table2 in the method section.

Validation

All antibodies were obtained from commercial sources and were validated by the company; refer to the company website for detailed validation analysis. The antibodies were also validated in our laboratory by ChIP-seq (specific peaks), western blots (one band corresponding to the expected size), and immuno-fluorescence (nuclear staining in ES cells).

Eukaryotic cell lines

Policy information about cell lines and Sex and Gender in Research

Cell line source(s)

Mouse embryonic fibroblasts (MEFs) were derived from 129 strain mice kept at the University of Edinburgh animal facility.
HEK 293T cell lines were used for lentivirus production (TAKARA #63218). These cells are isolated from human embryonic kidneys (HEK) and the 293T cells are transformed with large T antigen. HEK 293T cell line was originally created in Michele Calos's lab at Stanford (DuBridge et al; doi:10.1128/MCB.7.1.379).
Mouse iPSCs, iTSCs, ESCs, and TSCs were all generated in Yossi Buganim's laboratory in the Hebrew University and Hadassah Medical Center.

Authentication

MEFs were authenticated by the University of Edinburgh Animal facility by genotyping DNA extracted from the tail clip or ear notch using PCR. Mouse iPSCs, iTSCs, ESCs, and TSCs and their derivatives were authenticated by PCR and immunofluorescence in Yossi Buganim's laboratory as previously reported (Benchetrit et al; DOI: 10.1016/j.stem.2019.03.018).
The original HEK 293T cell line was authenticated previously (DuBridge et al; doi:10.1128/MCB.7.1.379). The commercial Lenti-X 293T Cell Line used in this study is a human embryonic kidney (HEK) cell line, transformed with adenovirus type 5 DNA, that also expresses the SV40 large T antigen. The cell line was subcloned for high transfectability and high-titer virus production by TAKARA (JAPAN). This lot of cells has been tested and found to be free of Mycoplasma contamination by TAKARA.

Mycoplasma contamination

All cells were routinely checked for Mycoplasma contamination and tested negative.

Commonly misidentified lines
(See ICLAC register)

No misidentified cell lines were used in this study.

Animals and other research organisms

Policy information about studies involving animals; ARRIVE guidelines recommended for reporting animal research, and Sex and Gender in Research

Laboratory animals

Blastocyst were derived from mouse CB6F1 host females after mating with CB6F1 males. Injected blastocysts were transferred to 2.5dpc pseudo-pregnant CD1/ICR females.

Wild animals

no wild animals were used in the study.

Reporting on sex	Sex information has not been collected and no sex- or gender-based analysis have been carried out as it is not relevant to this study.
Field-collected samples	no field collected samples were used in the study.
Ethics oversight	All animal experiments for the iPSC and iTSC generation from mouse embryonic fibroblasts were approved by the University of Edinburgh Animal Welfare and Ethical Review Body, performed at the University of Edinburgh, and carried out according to regulations specified by the Home Office and Project License. All reprogramming experiments have been approved by the University of Edinburgh SBS ethics committee (asoufi-0001). The joint ethics committee (IACUC) of the Hebrew University and Hadassah Medical Center approved the study protocol for animal welfare. The Hebrew University is an AAALAC international accredited institute. This research was performed in compliance with the Ethic Committee of Shaare Zedek Medical Center, the joint ethics committee (IACUC) of the Hebrew University and Hadassah Medical Center and the National ethic committee (Israel health ministry) and NIH.

Note that full information on the approval of the study protocol must also be provided in the manuscript.

Plants

Seed stocks	N/A
Novel plant genotypes	N/A
Authentication	N/A

ChIP-seq

Data deposition

☒ Confirm that both raw and final processed data have been deposited in a public database such as GEO.

☒ Confirm that you have deposited or provided access to graph files (e.g. BED files) for the called peaks.

Data access links<br>*May remain private before publication.*	To review GEO accession GSE201852:<br>Go to https://www.ncbi.nlm.nih.gov/geo/query/acc.cgi?acc=GSE201852<br>Enter token izkngkyynzutvmv into the box
Files in database submission	GSM6077123 Oct4-ChIP-seq, mESCs, biol rep 1<br>GSM6077124 Oct4-ChIP-seq, mESCs, biol rep 2<br>GSM6077125 Sox2-ChIP-seq, mESCs, biol rep 1<br>GSM6077126 Sox2-ChIP-seq, mESCs, biol rep 2<br>GSM6077127 Klf4-ChIP-seq, mESCs, biol rep 1<br>GSM6077128 Klf4-ChIP-seq, mESCs, biol rep 2<br>GSM6077129 Myc-ChIP-seq, mESCs, biol rep 1<br>GSM6077130 Myc-ChIP-seq, mESCs, biol rep 2<br>GSM6077131 Input-ChIP-seq, mESCs, biol rep 1<br>GSM6077132 Input-ChIP-seq, mESCs, biol rep 2<br>GSM6077133 Input-ChIP-seq, miTSCs, biol rep 1<br>GSM6077134 Input-ChIP-seq, miTSCs, biol rep 2<br>GSM6077135 Gata3-ChIP-seq, miTSCs, biol rep 1<br>GSM6077136 Gata3-ChIP-seq, miTSCs, biol rep 2<br>GSM6077137 Eomes-ChIP-seq, miTSCs, biol rep 1<br>GSM6077138 Eomes-ChIP-seq, miTSCs, biol rep 2<br>GSM6077139 Tfap2c-ChIP-seq, miTSCs, biol rep 1<br>GSM6077140 Tfap2c-ChIP-seq, miTSCs, biol rep 2<br>GSM6077141 Myc-ChIP-seq, miTSCs, biol rep 1<br>GSM6077142 Myc-ChIP-seq, miTSCs, biol rep 2<br>GSM6077143 Sox2-ChIP-seq, miTSCs, biol rep 1<br>GSM6077144 Sox2-ChIP-seq, miTSCs, biol rep 2<br>GSM6077145 Oct4-ChIP-seq, OSKM-48h, biol rep 1<br>GSM6077146 Oct4-ChIP-seq, OSKM-48h, biol rep 2<br>GSM6077147 Sox2-ChIP-seq, OSKM-48h, biol rep 1<br>GSM6077148 Sox2-ChIP-seq, OSKM-48h, biol rep 2<br>GSM6077149 Klf4-ChIP-seq, OSKM-48h, biol rep 1<br>GSM6077150 Klf4-ChIP-seq, OSKM-48h, biol rep 2<br>GSM6077151 Myc-ChIP-seq, OSKM-48h, biol rep 1<br>GSM6077152 Myc-ChIP-seq, OSKM-48h, biol rep 2<br>GSM6077153 Input-ChIP-seq, OSKM-48h, biol rep 1

GSM6077154 Input-ChIP-seq, OSKM-48h, biol rep 2
GSM6077155 Gata3-ChIP-seq, GETM-48h, biol rep 1
GSM6077156 Gata3-ChIP-seq, GETM-48h, biol rep 2
GSM6077157 Eomes-ChIP-seq, GETM-48h, biol rep 1
GSM6077158 Eomes-ChIP-seq, GETM-48h, biol rep 2
GSM6077159 Tfap2c-ChIP-seq, GETM-48h, biol rep 1
GSM6077160 Tfap2c-ChIP-seq, GETM-48h, biol rep 2
GSM6077161 Myc-ChIP-seq, GETM-48h, biol rep 1
GSM6077162 Myc-ChIP-seq, GETM-48h, biol rep 2
GSM6077163 Input-ChIP-seq, GETM-48h, biol rep 1
GSM6077164 Input-ChIP-seq, GETM-48h, biol rep 2
GSM6077165 Gata3-ChIP-seq, GETMR-48h, biol rep 1
GSM6077166 Gata3-ChIP-seq, GETMR-48h, biol rep 2
GSM6077167 Eomes-ChIP-seq, GETMR-48h, biol rep 1
GSM6077168 Eomes-ChIP-seq, GETMR-48h, biol rep 2
GSM6077169 Tfap2c-ChIP-seq, GETMR-48h, biol rep 1
GSM6077170 Tfap2c-ChIP-seq, GETMR-48h, biol rep 2
GSM6077171 Myc-ChIP-seq, GETMR-48h, biol rep 1
GSM6077172 Myc-ChIP-seq, GETMR-48h, biol rep 2
GSM6077173 Esrrb-ChIP-seq, GETMR-48h, biol rep 1
GSM6077174 Esrrb-ChIP-seq, GETMR-48h, biol rep 2
GSM6077175 Input-ChIP-seq, GETMR-48h, biol rep 1
GSM6077176 Input-ChIP-seq, GETMR-48h, biol rep 2
GSM6077177 Gata4-ChIP-seq, BS9G4M-48h, biol rep 1
GSM6077178 Gata4-ChIP-seq, BS9G4M-48h, biol rep 2
GSM6077179 Brn2-ChIP-seq, BS9G4M-48h, biol rep 1
GSM6077180 Brn2-ChIP-seq, BS9G4M-48h, biol rep 2
GSM6077181 Sox9-ChIP-seq-BS9G4M48h_rep1
GSM6077182 Sox9-ChIP-seq, BS9G4M-48h, biol rep 2
GSM6077183 Myc-ChIP-seq, BS9G4M-48h, biol rep 1
GSM6077184 Myc-ChIP-seq, BS9G4M-48h, biol rep 2
GSM6077185 Input-ChIP-seq, BS9G4M-48h, biol rep 1
GSM6077186 Input-ChIP-seq, BS9G4M-48h, biol rep 2
GSM6077187 Esrrb-ChIP-seq, mESCs
GSM6077188 Esrrb-ChIP-seq, miTSCs
GSM6077189 1U MNase, MEFs
GSM6077190 4U MNase, MEFs
GSM6077191 16U MNase, MEFs
GSM6077192 64U MNase, MEFs
GSM6077193 1U MNase, mESCs
GSM6077194 4U MNase, mESCs
GSM6077195 16U MNase, mESCs
GSM6077196 64U MNase, mESCs
GSM6077197 1U MNase, mTSCs
GSM6077198 4U MNase, mTSCs
GSM6077199 16U MNase, mTSCs
GSM6077200 64U MNase, mTSCs
GSM6077201 15U Mnase MicroC, MEFs
GSM6077202 20U Mnase MicroC, MEFs
GSM6077203 15U Mnase MicroC, mESCs
GSM6077204 20U Mnase MicroC, mESCs
GSM6077205 15U Mnase MicroC, mTSCs
GSM6077206 20U Mnase MicroC, mTSCs
GSM8351362 15U Mnase MicroC, MEF-H1OE
GSM8351363 20U Mnase MicroC, MEF-H1OE
GSM8351364 15U Mnase MicroC, MEF-H1KD
GSM8351365 20U Mnase MicroC, MEF-H1KD
GSM8354076 MEF, ATAC-seq, rep_1
GSM8354077 MEF, ATAC-seq, rep_2
GSM8354078 MEF, empty vector control, ATAC-seq, rep_1
GSM8354079 MEF, empty vector control, ATAC-seq, rep_2
GSM8354080 MEF, empty vector control, ATAC-seq, rep_3
GSM8354081 MEF, empty vector control, ATAC-seq, rep_4
GSM8354082 MEF, H1KD, ATAC-seq, rep_1
GSM8354083 MEF, H1KD, ATAC-seq, rep_2
GSM8354084 MEF, H1KD, ATAC-seq, rep_3
GSM8354085 MEF, H1KD, ATAC-seq, rep_4
GSM8354086 MEF, H1OE, ATAC-seq, rep_1
GSM8354087 MEF, H1OE, ATAC-seq, rep_2
GSM8354088 MEF, H1OE, ATAC-seq, rep_3
GSM8354089 MEF, H1OE, ATAC-seq, rep_4
GSM8354090 MKOS_MEF, 0h, ATAC-seq, rep_1
GSM8354091 MKOS_MEF, 0h, ATAC-seq, rep_2
GSM8354092 MKOS_MEF, 72h, ATAC-seq, rep_1
GSM8354093 MKOS_MEF, 72h, ATAC-seq, rep_2
GSM8354094 MKOS_MEF, 0h, H1KD, ATAC-seq, rep_1

GSM8354095 MKOS_MEF, 0h, H1KD, ATAC-seq, rep_2
GSM8354096 MKOS_MEF, 72h, H1KD, ATAC-seq, rep_1
GSM8354097 MKOS_MEF, 72h, H1KD, ATAC-seq, rep_2
GSM8354098 MKOS_MEF, 72h, H1OE, ATAC-seq, rep_1
GSM8354099 MKOS_MEF, 72h, H1OE, ATAC-seq, rep_2
GSM8354100 MKOS_MEF, 0h, empty_ctrl, ATAC-seq, rep_1
GSM8354101 MKOS_MEF, 0h, empty_ctrl, ATAC-seq, rep_2
GSM8354102 MKOS_MEF, 72h, empty_ctrl, ATAC-seq, rep_1
GSM8354103 MKOS_MEF, 72h, empty_ctrl, ATAC-seq, rep_2

Genome browser session
(e.g. UCSC)

Following normalized files have been submitted in GEO, which can be used in genome browser:

GSE201852_Brn2-ChIP-seq_BS9G4M48h.SeqDepthNorm.bw 331.8 Mb (http) BW
GSE201852_Eomes-ChIP-seq_GETM48h.SeqDepthNorm.bw 321.9 Mb (http) BW
GSE201852_Eomes-ChIP-seq_GETMR48h.SeqDepthNorm.bw 345.4 Mb (http) BW
GSE201852_Eomes-ChIP-seq_miTSC.SeqDepthNorm.bw 257.9 Mb (http) BW
GSE201852_Esrrb-ChIP-seq_GETMR48h.SeqDepthNorm.bw 374.5 Mb (http) BW
GSE201852_Esrrb-ChIP-seq_mESC.SeqDepthNorm.bw 259.6 Mb (http) BW
GSE201852_Esrrb-ChIP-seq_miTSC.SeqDepthNorm.bw 331.3 Mb (http) BW
GSE201852_Gata3-ChIP-seq_GETM48h.SeqDepthNorm.bw 310.4 Mb (http) BW
GSE201852_Gata3-ChIP-seq_GETMR48h.SeqDepthNorm.bw 306.6 Mb (http) BW
GSE201852_Gata3-ChIP-seq_miTSC.SeqDepthNorm.bw 287.2 Mb (http) BW
GSE201852_Gata4-ChIP-seq_BS9G4M48h.SeqDepthNorm.bw 249.1 Mb (http) BW
GSE201852_Input-ChIP-seq_BS9G4M48h.SeqDepthNorm.bw 356.4 Mb (http) BW
GSE201852_Input-ChIP-seq_GETM48h.SeqDepthNorm.bw 357.4 Mb (http) BW
GSE201852_Input-ChIP-seq_GETMR48h.SeqDepthNorm.bw 353.5 Mb (http) BW
GSE201852_Input-ChIP-seq_OSKM48h.SeqDepthNorm.bw 390.1 Mb (http) BW
GSE201852_Input-ChIP-seq_mESC.SeqDepthNorm.bw 491.4 Mb (http) BW
GSE201852_Input-ChIP-seq_miTSC.SeqDepthNorm.bw 507.2 Mb (http) BW
GSE201852_Klf4-ChIP-seq_OSKM48h.SeqDepthNorm.bw 321.6 Mb (http) BW
GSE201852_Klf4-ChIP-seq_mESC.SeqDepthNorm.bw 326.2 Mb (http) BW
GSE201852_MEF-H1KD_combined_MicroC.mm9.mapq_30.100.mcool 3.1 Gb (http) MCOOL
GSE201852_MEF-H1OE_combined_MicroC.mm9.mapq_30.100.mcool 2.4 Gb (http) MCOOL
GSE201852_MEF_H1KD_merged_SeqDepthNorm.bw 448.5 Mb (http) BW
GSE201852_MEF_H1OE_merged_SeqDepthNorm.bw 420.0 Mb (http) BW
GSE201852_MEF_combined_MicroC.mm9.mapq_30.100.mcool 2.2 Gb (http) MCOOL
GSE201852_MEF_empty_merged_SeqDepthNorm.bw 426.5 Mb (http) BW
GSE201852_MEF_merged_SeqDepthNorm.bw 235.0 Mb (http) BW
GSE201852_MKOS_MEF_0hr_merged_SeqDepthNorm.bw 266.1 Mb (http) BW
GSE201852_MKOS_MEF_72hr_merged_SeqDepthNorm.bw 283.7 Mb (http) BW
GSE201852_MKOS_MEF_H1KD_0hr_merged_SeqDepthNorm.bw 170.7 Mb (http) BW
GSE201852_MKOS_MEF_H1KD_72hr_merged_SeqDepthNorm.bw 156.2 Mb (http) BW
GSE201852_MKOS_MEF_H1OE_72hr_merged_SeqDepthNorm.bw 392.3 Mb (http) BW
GSE201852_MKOS_MEF_empty_0hr_merged_SeqDepthNorm.bw 181.4 Mb (http) BW
GSE201852_MKOS_MEF_empty_72hr_merged_SeqDepthNorm.bw 224.8 Mb (http) BW
GSE201852_Myc-ChIP-seq_BS9G4M48h.SeqDepthNorm.bw 233.3 Mb (http) BW
GSE201852_Myc-ChIP-seq_GETM48h.SeqDepthNorm.bw 292.1 Mb (http) BW
GSE201852_Myc-ChIP-seq_GETMR48h.SeqDepthNorm.bw 253.1 Mb (http) BW
GSE201852_Myc-ChIP-seq_OSKM48h.SeqDepthNorm.bw 286.3 Mb (http) BW
GSE201852_Myc-ChIP-seq_mESC.SeqDepthNorm.bw 318.1 Mb (http) BW
GSE201852_Myc-ChIP-seq_miTSC.SeqDepthNorm.bw 378.6 Mb (http) BW
GSE201852_Oct4-ChIP-seq_OSKM48h.SeqDepthNorm.bw 361.7 Mb (http) BW
GSE201852_Oct4-ChIP-seq_mESC.SeqDepthNorm.bw 338.9 Mb (http) BW
GSE201852_Sox2-ChIP-seq_OSKM48h.SeqDepthNorm.bw 432.3 Mb (http) BW
GSE201852_Sox2-ChIP-seq_mESC.SeqDepthNorm.bw 333.2 Mb (http) BW
GSE201852_Sox2-ChIP-seq_miTSC.SeqDepthNorm.bw 251.6 Mb (http) BW
GSE201852_Sox9-ChIP-seq_BS9G4M48h.SeqDepthNorm.bw 301.4 Mb (http) BW
GSE201852_Tfap2c-ChIP-seq_GETM48h.SeqDepthNorm.bw 311.3 Mb (http) BW
GSE201852_Tfap2c-ChIP-seq_GETMR48h.SeqDepthNorm.bw 376.4 Mb (http) BW
GSE201852_Tfap2c-ChIP-seq_miTSC.SeqDepthNorm.bw 228.4 Mb (http) BW
GSE201852_mESC_combined_MicroC.mm9.mapq_30.100.mcool 2.7 Gb (http) MCOOL
GSE201852_mTSC_combined_MicroC.mm9.mapq_30.100.mcool 2.4 Gb (http) MCOOL

Methodology

Replicates

Three ChIP replicates were pooled to make a DNA library for each ChIP-seq experiment and two independent replicates were carried out. All other sequencing data were carried out in duplicates or a pool of triplicates. Different MNase concentrations were used to generate independent sequencing libraries.

Sequencing depth

Around 50-60 million pair-end reads were obtained on average from each ChIP-seq.

Antibodies

Listed in Table 2 of the method section.

Peak calling parameters

Duplicates were removed from the aligned pair-end BAM files using Picard prior to peak calling. TF peaks (sample files) showing significant enrichment over input DNA (control files) obtained from the same cells were called using MACS2 (version 2.1.1.20160309)

	and a fragment size of 200 bp (--nomodel --extsize 200) and were controlled to q value (minimum FDR) cut-off of 0.01 (-q 0.01). The peaks that overlapped with the ENCODE mm9 blacklist were removed using the bedtools intersect function (flag –v).<br>To identify broadPeaks of TF binding, peaks were called as using MACS2 with the following flags: -B --broad-cutoff 0.1 --broad --nomodel --extsize 200. Regions that overlapped with the ENCODE blacklist were removed using the bedtools intersect function (flag –v).
Data quality	Quality controls of DNA libraries were carried out by DNA fragment size distribution using Tapestation and Bioanalyzer (Agilent). Sequencing quality was assessed by mean quality scores using FASTQC and only Phred scores above 30 were considered. Sequence duplication and library complexity was assessed by MutiQC and Qualimap prior to further analysis. Duplicates were removed by Picard and adapters by Cutadapt. Libraries were normalized by squencing depth to 1X genome coverage using DeepTools.
Software	FASTQC v0.11.8, MultiQC v1.3, Bowtie v2.3, Bedtools V2.28, Picard v2.20, MACS v2.1.1, DeepTools V2, Qualimap V2.2.1, SAMTool2 v1.3.1, MEME v5.0.2, R v3.6 (various R packages as indicated in the methods), BEDOPS V2.49 and Cutadapt v3.3.

Flow Cytometry

Plots

Confirm that:

☒ The axis labels state the marker and fluorochrome used (e.g. CD4-FITC).

☒ The axis scales are clearly visible. Include numbers along axes only for bottom left plot of group (a 'group' is an analysis of identical markers).

☒ All plots are contour plots with outliers or pseudocolor plots.

☒ A numerical value for number of cells or percentage (with statistics) is provided.

Methodology

Sample preparation	cells were first trypsinized and then neutralized with medium containing 10% fetal bovine serum (FBS). Following this, the cells were centrifuged and washed twice with phosphate-buffered saline (PBS) to ensure the removal of any residual trypsin and medium. The washed cells were then resuspended in PBS for subsequent analysis.
Instrument	Flow cytometric analysis was performed using a Beckman Coulter (Gallios) flow cytometer.
Software	Data acquisition and analysis were conducted using the Kaluza Software (Version 1.0.14029.14028).
Cell population abundance	The fluorescent markers eGFP and tdTomato were used to identify and quantify specific cell populations.
Gating strategy	To remove dead cells, all samples were initially gated using the FSC-A/SSC-A gating to identify the live cell population (below 200 FS Area). To remove cell doublets, single cells were selected by gating forward scatter height vs area. The positively fluorescent cells were gated based on the fluorescent intensity of positive control cells.

☒ Tick this box to confirm that a figure exemplifying the gating strategy is provided in the Supplementary Information.
